# Interaction of ERα and NRF2 Impacts Survival in Ovarian Cancer Patients

**DOI:** 10.3390/ijms20010112

**Published:** 2018-12-29

**Authors:** Bastian Czogalla, Maja Kahaly, Doris Mayr, Elisa Schmoeckel, Beate Niesler, Thomas Kolben, Alexander Burges, Sven Mahner, Udo Jeschke, Fabian Trillsch

**Affiliations:** 1 Department of Obstetrics and Gynecology, University Hospital, LMU Munich, 81377 Munich, Germany; bastian.czogalla@med.uni-muenchen.de (B.C.); maja.kahaly@googlemail.com (M.K.); thomas.kolben@med.uni-muenchen.de (T.K.); alexander.burges@med.uni-muenchen.de (A.B.); sven.mahner@med.uni-muenchen.de (S.M.); fabian.trillsch@med.uni-muenchen.de (F.T.); 2 Institute of Pathology, Faculty of Medicine, 81377 LMU Munich, Germany; doris.mayr@med.uni-muenchen.de (D.M.); elisa.schmoeckel@med.uni-muenchen.de (E.S.); 3 Institute of Human Genetics, Department of Human Molecular Genetics, University of Heidelberg, 69120 Heidelberg, Germany; Beate.Niesler@med.uni-heidelberg.de

**Keywords:** estrogen receptor alpha, nuclear factor erythroid 2-related factor 2, ovarian cancer, immunohistochemistry

## Abstract

Nuclear factor erythroid 2-related factor 2 (NRF2) regulates cytoprotective antioxidant processes. In this study, the prognostic potential of NRF2 and its interactions with the estrogen receptor α (ERα) in ovarian cancer cells was investigated. NRF2 and ERα protein expression in ovarian cancer tissue was analyzed as well as mRNA expression of NRF2 (*NFE2L2*) and ERα (*ESR1*) in four ovarian cancer and one benign cell line. *NFE2L2* silencing was carried out to evaluate a potential interplay between NRF2 and ERα. Cytoplasmic NRF2 expression as inactive form had significantly higher expression in patients with low-grade histology (*p* = 0.03). In the serous cancer subtype, high cytoplasmic NRF2 expression (overall survival (OS), median 50.6 vs. 29.3 months; *p* = 0.04) and high ERα expression (OS, median 74.5 vs. 27.1 months; *p* = 0.002) was associated with longer overall survival as well as combined expression of both inactive cytoplasmic NRF2 and ERα in the whole cohort (median 74.5 vs. 37.7 months; *p* = 0.04). Cytoplasmic NRF2 expression showed a positive correlation with ERα expression (*p* = 0.004). *NFE2L2* was found to be highly expressed in the ovarian cancer cell lines OVCAR3, UWB1.289, and TOV112D. Compared with the benign cell line HOSEpiC, *ESR1* expression was reduced in all ovary cancer cell lines (all *p* < 0.001). Silencing of *NFE2L2* induced a higher mRNA expression of *ESR1* in the *NFE2L2* downregulated cancer cell lines OVCAR3 (*p* = 0.003) and ES2 (*p* < 0.001), confirming genetic interactions of NRF2 and ERα. In this study, both inactive cytoplasmic NRF2 and high ERα expression were demonstrated to be associated with improved survival in ovarian cancer patients. Further understanding of interactions within the estradiol–ERα–NRF2 pathway could better predict the impact of endocrine therapy in ovarian cancer.

## 1. Introduction

Ovarian cancer is the eighth most frequent cause of cancer death among women and the most lethal gynecological malignancy [1]. Relative five-year survival is less than 50% for patients with epithelial ovarian carcinoma (EOC) [2]. Main reasons for poor prognosis are insufficient screening methods, late stage detection, and resistance to chemotherapy later in the clinical course. As most patients have advanced stage disease, recommended therapy consists of cytoreductive surgery and platinum-based chemotherapy which might be combined with antiangiogenic bevacizumab. Residual disease after initial debulking surgery is the most important prognostic factor being influenced by treating physicians, while further clinical and pathological prognostic factors include the degree of differentiation, the International Federation of Gynecology and Obstetrics (FIGO) stage, and histological subtype [3,4,5,6]. With serous, mucinous, endometrioid, and clear cell histology, invasive EOC exhibits several histopathological subtypes that are phenotypically, molecularly, and etiologically distinct [7]. The association between tumor biomarker expression and survival varies substantially between subtypes and can be distinguished in overall analyses of all EOCs [8,9].

According to current investigations, the occurrence of EOC seems to be related to oxidative stress [9]. By activating the nuclear factor erythroid-2-related factor 2 (NRF2), a relevant regulator of antioxidant and cytoprotective genes, both healthy and tumor cells can cope with oxidative stress. NRF2 is ubiquitously expressed at low levels in all human organs. As NRF2 regulates a major cellular defense mechanism, tight regulation is crucial to maintain cellular homeostasis. High constitutive levels of NRF2 have been described in different tumors or cancer cell lines [10,11,12,13,14]. Overexpression of NRF2 might protect cancer cells from the cytotoxic effects of anticancer therapies, resulting in resistance for chemo- or radiotherapy [15,16].

So far, the role of estrogen in EOC is still debated [17]. While application of exogenous hormones for menopause-related symptoms could be associated with an increased risk of EOC [18], a protective effect of oral contraceptives has been described. The estrogen receptor (ER) is expressed in two isoforms, the ERα and ERß [19]. ERα mediates the effects of female steroid hormones on proliferation and apoptosis of EOC cells, and immunohistochemical assessment of ER status is routinely done for the clinical management of breast cancer [19]. Molecular and cell biological interactions between NRF2 and ERα have been reported so far [16,20]. The aryl hydrocarbon receptor and ERα differentially modulate NRF2 transactivation in MCF-7 breast cancer cells [16]. Furthermore, studies show an important crosstalk between NRF2 and ERα in neurophysiological processes [16,20].

To better understand these effects in EOC, we first assessed the prognostic influence of NRF2 and ERα in various subtypes of EOC. To understand the interaction of NRF2 and Erα on a molecular level, we investigated the expression and their correlation in vitro.

## 2. Results

### 2.1. NRF2/ERα Expression Correlates with Cinical and Pathological Data

**Figure 1:**
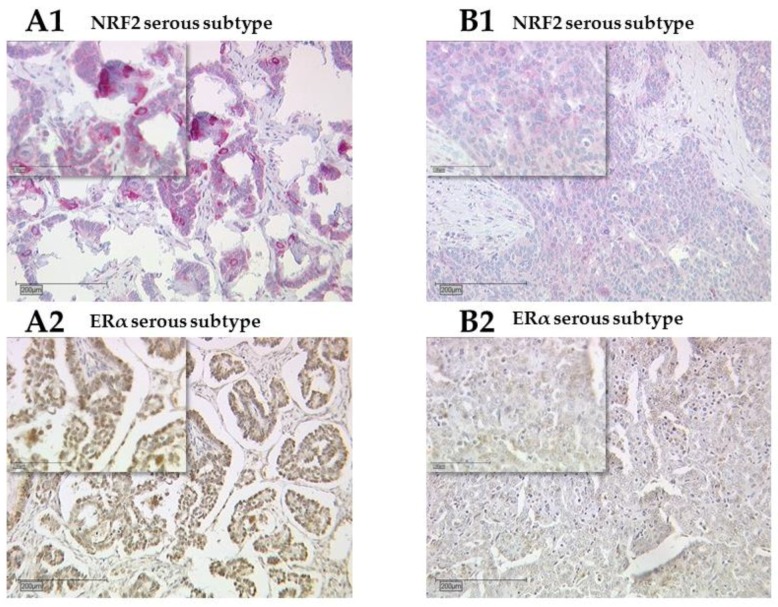
Detection of nuclear factor erythroid-2-related factor 2 (NRF2) (**A1, B1**) and estrogen receptor (ER)α (**A2, B2**) with immunohistochemistry. High (**A1**) and low (**B1**) cytoplasmic NRF2 stains in serous subtype correspond with high (**A2**) and low (**B2**) ERα stains, respectively. NRF2 shows faint staining in the nucleus in both cases (**A1, B1**).

Nuclear staining of NRF2 was technically successful in 145 of 156 cases (93%) with positive staining in 144 of 145 cases (99%). Cytoplasmic staining of NRF2 was evaluable with technically adequate staining in 139 of 156 cases (89%) (Figure 1 and Figures S1 and S2) and NRF2 expression was observed in all these 139 specimens (100%). Median (range) immunoreactivity scores (IRS) for NRF2 in nuclei and cytoplasm were 8 (2,12) and 8 (4,12), respectively. 

NRF2 expression displayed correlations to clinical and pathological data (Table 1). NRF2 staining in both cytoplasm and nucleus was different between the histological subtypes (*p* = 0.001 and *p* = 0.02, respectively) with low nuclear NRF2 expression in serous, clear cell, and endometrioid histology and high expression in the mucinous subtype. In comparison, the strongest and weakest cytoplasmic NRF2 staining was found in the serous and clear cell subtypes, respectively. Cytoplasmic NRF2 expression had significantly higher expression in patients with low-grade histology (*p* = 0.03), and low nuclear NRF2 expression was associated with age (*p* = 0.045) (Table 1). 

**Figure 2:**
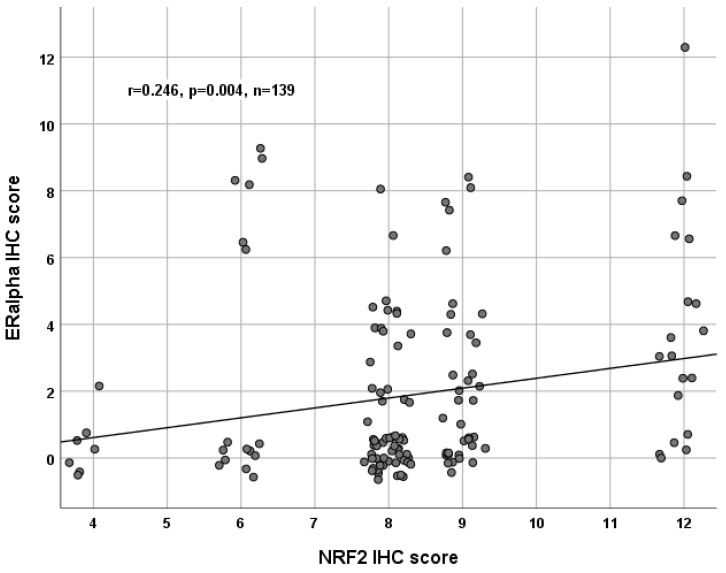
Correlation analysis of NRF2 and ERα in ovarian cancer tissue (*n* = 139). A significant correlation of cytoplasmic NRF2 expression with ERα expression was noted. For better visualization, dots have been jittered.

ERα staining was successfully performed in all 156 cases (100%), and ERα expression was observed in 70 of 156 (45%) specimens with a median (range) IRS of 4 (1,12) (Figure 1 and Figure S1). There was no significant difference in the ERα expression comparing all histological subtypes (*p* = 0.21). Analysis of clear cell and endometrioid ovarian cancer subtypes revealed nearly significant upregulation (*p* = 0.05). Analyzing the grading, there were no significant differences in general, and low-graded patients showed significantly higher ERα expression compared to high-graded patients (*p* = 0.028). All other parameters, such as FIGO, lymph node involvement (pN), and distant metastasis (pM), showed no significant differences in the ERα expression. NRF2 cytoplasmic expression correlated with ERα expression (*p* = 0.004, Table 2 and Figure 1 and Figure 2).

### 2.2. High NRF2/ERα Expression is Associated with Improved Overall Survival 

**Figure 3:**
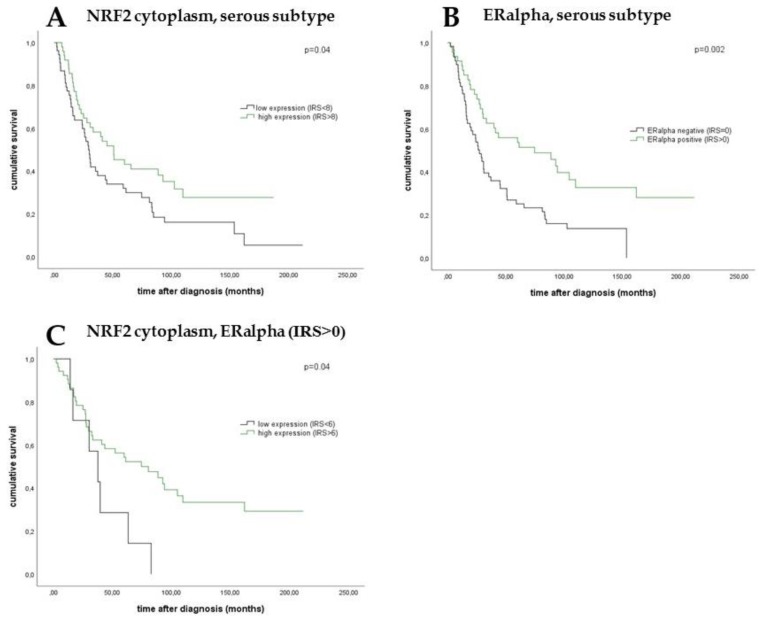
Kaplan–Meier estimates of NRF2 expression, ERα expression, and combined NRF2 and ERα expression were analyzed. In the serous subtype, patients with a high cytoplasmic expression of NRF2 showed a significantly increased overall survival compared with patients with a low cytoplasmic expression (**A**). In addition, high ERα expression was associated with significantly better overall survival in serous ovarian cancer compared with patients with a low ERα expression (**B**). Patients with combined high NRF2 expression in the cytoplasm and ERα expression in epithelial ovarian carcinoma (EOC) had significantly increased overall survival compared with those with low cytoplasmic expression and ERα expression (**C**).

The median age of the patients was 58.7 (standard deviation (SD) of 31.4) years with a range of 31–88 years. Median overall survival of the EOC patients was 34.4 (SD 57.8) months. Cytoplasmic NRF2 expression in the serous cancer subtype was associated with longer overall survival (Figure 3, median 50.6 vs. 29.3 months; *p* = 0.04) as it was noted for ERα expression (Figure 3, median 74.5 vs. 27.1 months; *p* = 0.002). Improved OS was also seen for patients with combined and high expression of both NRF2 and ERα in the cytoplasm comparing all histological subtypes (Figure 3, median 74.5 vs. 37.7 months; *p* = 0.04).

### 2.3. Clinical and Pathological Parameters are Independent Prognostic Factors

Cancer grading, the FIGO classification, and patients’ age were independent prognostic factors in the present cohort (Table 3). In contrast, prognostic impact of histological subtype, NRF2, and/or ERα staining/expression was not significant.

### 2.4. Downregulation of NFE2L2 Increases ESR1 Expression, Confirming Their Genetic Interaction

**Figure 4:**
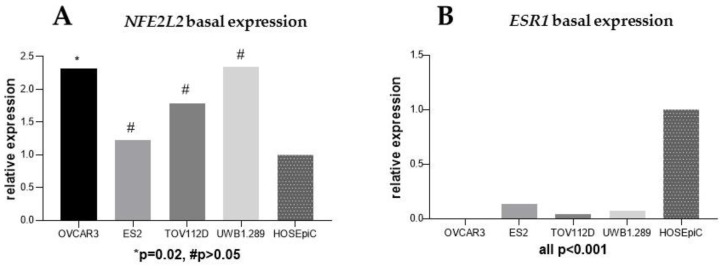
Basal gene expression of *NFE2L2* (**A**) and *ESR1* (**B**) in four ovarian cancer cell lines was compared to the expression in the benign ovarian cell line (HOSEpiC).

Basal expressions of both *NFE2L2* and *ESR1* were analyzed by qPCR in all four EOC cell lines and compared with a benign ovarian cell line (HOSEpiC). As shown in Figure 4 and compared to HOSEpiC, *NFE2L2* expression increased 2-fold in both OVCAR3 (*p* = 0.02) and UWB1.289 (*p* = 0.08) and was 1.5-fold elevated in the TOV112D (*p* = 0.30) cell lines. In comparison, *ESR1* expression was markedly reduced in all EOC cell lines compared to the benign ovarian cells (all *p* < 0.001). 

**Figure 5:**
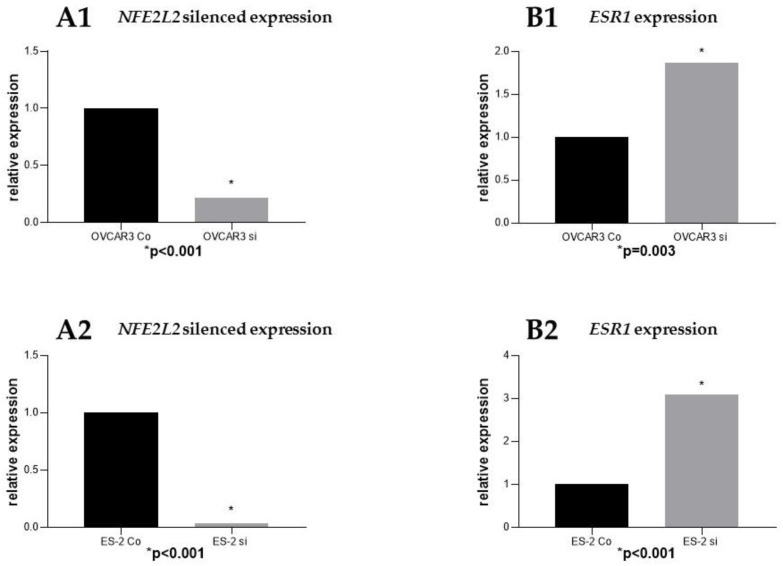
siRNA downregulation of *NFE2L2* in the ovarian cancer cell lines OVCAR3 (**A1**) and ES2 (**A2**). *ESR1* expression following *NFE2L2* downregulation in both cell lines (**B1, B2**).

Following effective silencing of *NFE2L2* with siRNA to evaluate the impact on *ESR1* expression (Figure 5), an elevated expression of *ESR1* in the *NFE2L2* downregulated cancer cell lines OVCAR3 (*p* = 0.003) and ES2 was noted (*p* < 0.001).

## 3. Discussion

This cell and molecular biological experimental study reveals that NRF2 expression differs in histologic subtypes of EOC, with the strongest cytoplasmic expression in the serous subtype. Cytoplasmic NRF2 expression had significantly higher expression in patients with low-grade histology. Patients with higher cytoplasmic NRF2 expression in the serous type confirmed to have a significantly improved OS. Moreover, we could reveal that the combination of cytoplasmic NRF2 and ERα expression was associated with significantly longer OS. Molecular testing in cell lines exhibited that the *ESR1* gene was lower expressed in all four EOC cell lines, which could be upregulated by *NFE2L2* silencing in the subsequently *NFE2L2*-downregulated cancer cell lines.

NRF2 has been traditionally considered as a tumor suppressor because its cytoprotective functions are deemed to be the main cellular defense mechanism against exogenous and endogenous insults, including xenobiotic and oxidative stress [21,22]. Under homeostatic conditions, NRF2 activation prevents excessive cellular damage produced by metabolic, xenobiotic, and oxidative stress [22]. NRF2 activation is thus important in cancer chemoprevention. Cancer chemoprevention mechanisms seem to be mediated through the Keap1–NRF2 pathway, and in experimental models, NRF2/Keap1 mutations are present at preneoplastic stages [23]. Further, NRF2-null mice are more prone to develop cancer in response to chemical and physical stimuli (nitrosamine, ultraviolet light, and aflatoxin) [17]. On the other hand, recent studies demonstrated that NRF2 hyperactivation may also create an environment favoring survival of normal as well as malignant cells, protecting them from apoptosis and senescence and against oxidative stress, chemotherapeutic agents, and radiotherapy [24,25]. Hence, the potential dual role of NRF2 in cancer may explain the described results below. 

**Figure 6:**
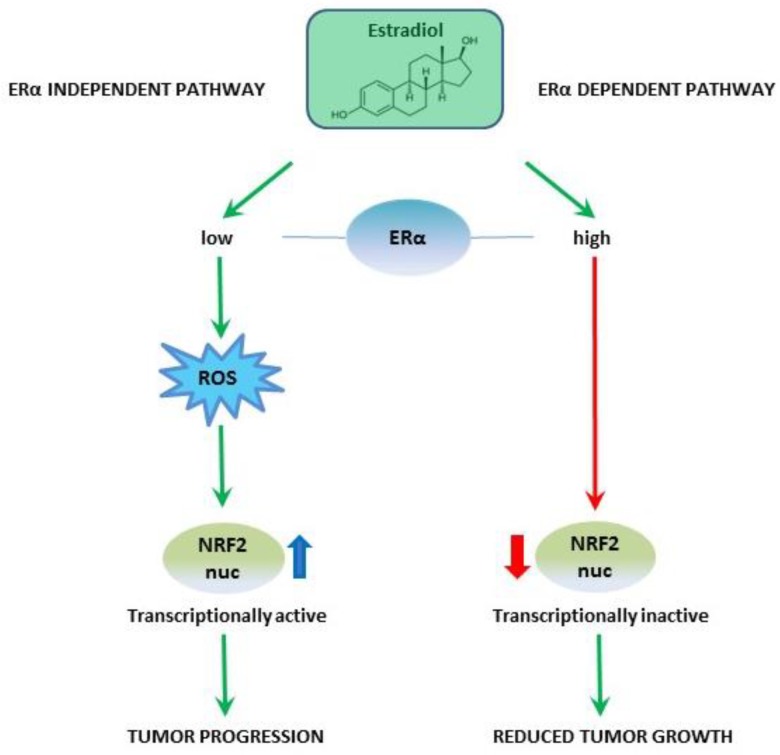
Summary of the hypothesized interaction within the estradiol–ERα–NRF2 pathway: High expression of ERα leads to an induction of the estradiol–ERα-dependent pathway, resulting in transcriptionally inactive NRF2 (low nuclear, high cytoplasmic expression) and consecutively less impact on tumor growth. In contrast, low ERα expression favors the estradiol–ERα-independent pathway, with activation of NRF2 (high nuclear, low cytoplasmic expression) causing tumor progression.

Our findings are in line with previous reports showing that nuclear or activated NRF2 expression is associated with upregulation of NRF2 target genes and poorer OS and disease-free survival (DFS), whereas patients with high cytoplasmic or inactive NRF2 expression displayed better OS and DFS [26]. Our evaluation of ER expression in the EOC tissue samples confirmed previous reports. In patients with EOC, the ER, especially ERα, is significantly associated with improved OS [8], grading, progression-free survival, and cause-specific survival, respectively [27]. There is a strong relationship between circulating sex hormones and female reproductive cancers (e.g., ovarian, breast, and endometrial cancers) [28]. Interestingly, estradiol may play a dual role in modulating NRF2 activity. On the one hand, its metabolites activate NRF2 via the generation of reactive oxygen species (ROS) (independent of the ER) [29]. While recent studies demonstrated that estradiol leads to an activation of NRF2 in a wide range of cell types [30,31], the estradiol effect was only noted on protein and not on mRNA levels, suggesting that the main effect of estradiol is based on NRF2 protein stabilization [32]. However, binding to ERα (dependent of ER) appears to be the mechanism for estradiol itself to inhibit the NRF2 downstream genes [9]. ERα, but not ERβ, interacts with NRF2 in an estradiol-dependent way and thereby represses NRF2-mediated transcription [33]. Thus, EOC patients with high tumor expression of ERα show a strong influence of the estradiol–ERα-dependent pathway, resulting in inactivated NRF2 and better survival rates. Otherwise, low ERα expression causes a dysbalance in favor of the estradiol–ERα-independent pathway with an activation of NRF2 (Figure 6). Studies show that other NRF2-associated factors also could play a crucial role in the above-described interaction. Glutathione S-transferase (GST), an NRF2 target gene, is modulated by miR-186 overexpression in OVCAR3 cells with consecutively increased sensitivity of ovarian cancer cells to paclitaxel [34]. Furthermore, it was described that the KEAP1–NRF2 pathway is important in ovarian cancer cell reaction to cigarette-smoke-induced ROS [35]. 

Endocrine therapy in EOC has been considered as a potential approach in subgroups of patients with a specific tumor biology that responds to this therapy [36]. Hereby, the rationale for endocrine treatment is based on the high ER/PR IHC expression as a predictive marker [37]. A present prospective study demonstrated evidence for the usefulness of letrozole as an aromatase inhibitor in serous EOC [38]. Under the conditions described above, treatment with aromatase inhibitors could cause a prognostically beneficial predominance of the ERα–NRF2-dependent pathway. As revealed in the present investigation, a putative functional association of endocrine therapy and NRF2 underlines the relationship of NRF2/ERα, as confirmed by significant correlation of expression. In addition to the mentioned approach, further therapeutic strategies as interference of DNA repair mechanisms are of great interest to overcome treatment burden [39,40,41,42].

The retrospective design, the relatively small number of tissue samples evaluated, and the semiquantitative scoring method may critically be regarded as limitations of the submitted work. The data are hypothesis generating and further prospective studies with a larger patient collective and standardized immunohistochemical and molecular methods are warranted to gain more detailed and better insight into this research field. 

However, despite these drawbacks, our analysis indicates for the first time a putative molecular role of the estradiol–ERα–NRF2 pathway as a basis for a better understanding of endocrine therapy in EOC.

## 4. Materials and Methods 

### 4.1. Ethical Approval

The current study was approved by the Ethics Committee of the Ludwig-Maximilians-University, Munich, Germany (approval number 227-09) on 30 September 2009. All tissue samples used for this study were obtained from material from the archives of LMU Munich, Department Gynecology and Obstetrics, Ludwig-Maximilians-University, Munich, Germany, initially used for pathological diagnostics. The diagnostic procedures were completed before the current study was performed. During the analysis, the observers were fully blinded to patients’ data. The study was approved by the Ethics Committee of LMU Munich. All experiments were performed according to the standards of the Declaration of Helsinki (1975).

### 4.2. Patients and Specimens

Tissue samples of 156 patients who underwent surgery for EOC at the Department of Obstetrics and Gynecology, Ludwig-Maximillian’s-University Munich from 1990 to 2002 were analyzed in this study. Clinical data was obtained from the patients’ charts and follow up data from the Munich Cancer Registry. All samples had been formalin-fixated and paraffin-embedded (FFPE). Patients with benign or borderline tumors were excluded and no patients had adjuvant chemotherapy. Specialized pathologists for EOC examined and classified the samples for tumor grading—low (*n* = 38), high (*n* = 117)—and histological subtypes—serous (*n* = 110), endometrioid (*n* = 21), clear cell (*n* = 12), and mucinous (*n* = 13). Staging was performed using TNM and FIGO (WHO) classification: I (*n* = 35), II (*n* = 10,) III (*n* = 103), and IV (*n* = 3). Data on primary tumor extension were available in 155 cases—T1 (*n* = 40), T2 (*n* = 18), T3 (*n* = 93), and T4 (*n* = 4)—as well as data on lymph node involvement in 95 cases—N0 (*n* = 43), N1 (*n* = 52). Data on distant metastasis were available in nine cases—M0 (*n* = 3), M1 (*n* = 6).

### 4.3. Immunohistochemistry

Immunohistochemistry was performed as previously described by our lab [43]. For NRF2 staining, FFPE EOC samples were incubated with anti-NRF2 (Abcam, Cambridge, UK, rabbit, monoclonal, clone EP1808) at a final concentration of 5.93 µg/ml (1:100 dilution) for 1 h at room temperature. Afterwards, slides were incubated with isotype-matching MACH 3 Rabbit AP Polymer Detection (Biocare Medical, Pacheco, CA, USA, catalogue-number M3R533). The Permanent AP Red Kit (Zytomed Systems GmbH, Berlin, Germany, catalogue-number ZUC-001) was used as a chromogen. Slides were then counterstained with Gill´s hematoxylin (Vector Laboratories, Burlingame, CA, USA). System controls were included. 

For the detection of ERα, resected EOC tissue samples were fixed in formalin and embedded in paraffin after surgery. ERα staining was performed by blocking slides with goat serum (1:100 dilution, Vectastain^®^ ABC-Elite-Kit, Linaris, Dossenheim, Germany, catalogue-number PK-6101) for 30 min at room temperature. Subsequently, slides were incubated with anti-ERα primary antibody (1:400 dilutions, Abcam, Cambridge, UK, rabbit, monoclonal, clone EPR703(2)) for 16 h at 4 °C. Afterwards, slides were incubated with isotype-matching anti-rabbit IgG secondary antibody and avidin–biotin–peroxidase complex both for 30 min at room temperature, according to the Vectastain^®^ ABC-Elite-Kit (Linaris, Dossenheim, Germany, catalogue-number PK-6101). All slides were washed twice in PBS for 2 min after every incubation step. 3,3’-Diaminobenzidine chromogen (DAB; Dako, Glostrup, Denmark, catalogue-number K3468) was used for visualization reaction. Slides were then counterstained with Mayer’s acidic hematoxylin (Waldeck-Chroma, Münster, Germany, catalogue number 2E-038) and dehydrated in an ascending series of alcohol followed by xylol. System controls were included.

### 4.4. Staining Evaluation and Statistical Analysis

All EOC specimens were examined with a Leitz (Wetzlar, Germany) photomicroscope and specific NRF2 and ERα immunohistochemical staining reaction was observed in the nuclei and cytoplasm of the cells. The intensity and distribution pattern of NRF2 and ERα staining was rated using the semiquantitative immunoreactivity score (IRS, Remmele’s score). To obtain the IRS result, the optional staining intensity (0 = no, 1 = weak, 2 = moderate, and 3 = strong staining) and the percentage of positive stained cells (0 = no staining, 1 = <10% of the cells, 2 = 11%–50% of the cells, 3 = 51%–80% of the cells, and 4 = >81%) were multiplied. Nuclear and cytoplasmic NRF2 staining was successfully performed in 145 (93%) and 139 (89%) of 156 EOC tissue specimens, respectively. Cut-off points for the IRSs were selected for cytoplasmic and nuclear NRF2 staining considering the distribution pattern of IRSs in the collective. Nuclear and cytoplasmic NRF2 staining were regarded as negative with an IRS of 0–2, as low with IRS of 4–8, and as high with IRS of ≥9. ERα staining was successfully performed in all 156 (100%) EOC specimens. Cellular ERα staining was considered as negative with an IRS of 0 and as positive with an IRS of >0. 

Statistical analysis was performed using SPSS 25.0 (v25, IBM, Armonk, New York). Distribution of clinical pathological variables was evaluated with the chi-squared test. The Mann–Whitney *U* test was used to compare IRSs of NRF2 between different clinical and pathological subgroups. Correlations between findings of immunohistochemical staining were calculated using Spearman’s analysis. Survival times were analyzed by Kaplan–Meier (log-rank) estimates. To identify an appropriate cut-off, the ROC curve was drawn, which is considered as one of the most reliable methods for cut-off point selection. In this context, the ROC curve was a plot representing sensitivity on the y-axis and (1-specificity) on x-axis [44]. Consecutively, Youden’s index, defined as the maximum (sensitivity+specificity-1) [45], was used to find the optimal cut-off maximizing the sum of sensitivity and specificity [46,47]. For multivariate analyses, a Cox regression model was applied, with *p*-values less than 0.05 considered to be significant. Ct values of each gene were obtained with qPCR and the relative expressions were calculated using the 2^−ΔΔCt^ formula. Statistical data was acquired using Graph Pad Prism 7.03 (v7, La Jolla, CA, USA).

### 4.5. Cell Lines

The human ovarian cancer cell lines OVCAR3 (serous), ES-2 (clear cell), TOV112D (endometrioid), and UWB1.289 (serous, BRCA1 negative) were purchased from the American Type Culture Collection (ATCC, Rockville, MD, USA). Cells were maintained in culture in RPMI 1640 medium (ThermoFisher Scientific, Waltham, MA, USA) supplemented with 10% FBS in a humidified incubator at 37 °C under 5% CO_2_. The benign ovarian cell line HOSEpiC was purchased from ScienCell (Carlsbad, CA, USA). HOSEpiC cells were maintained in culture in Ovarian Epithelial Cell Medium (OEpiCM) (ScienCell, Carlsbad, CA, USA, catalogue-number 7311) in a humidified incubator at 37 °C under 5% CO_2_.

### 4.6. PCR

RNA isolation was performed using the RNeasy Mini Kit (Qiagen, Venlo, Netherlands) and 1 µg of RNA was converted into first-strand cDNA using the MMLV Reverse Transcriptase 1st-Strand cDNA Synthesis Kit (Epicentre, Madison, WI, USA) according to the instructions of the manufacturer. The basal mRNA expressions of *NFE2L2* and *ESR1* were quantified by qPCR applying FastStart Essential DNA Probes Master and gene-specific primers (Roche, Basel, Switzerland). For normalization of expressions the housekeeping genes, *β-Actin* and *GAPDH* were used as reference controls. Basal expressions of *NFE2L2* and *ESR1* in the ovarian cancer cell lines were compared with their expressions in the benign ovarian cell lines.

### 4.7. siRNA

The specific siRNA for *NFE2L2* (Silencer Select Pre-designed and Custom Designed siRNA, Ambion, Carlsbad, CA, USA) was kindly provided by Beate Niesler (Department of Human Molecular Genetics, University of Heidelberg). Cells were transfected with siRNA using Lipofectamine RNAiMAX reagent (Invitrogen, Carlsbad, CA, USA) to silence the expression of *NFE2L2* in the cell lines. RNA isolation and mRNA quantification by qPCR was repeated as outlined above. mRNA expression levels of *NFE2L2* and *ESR1* in *NFE2L2*-downregulated cells were compared with *NFE2L2*-containing cells.

## 5. Conclusions

Here, *ESR1* expression was reduced in different ovarian cancer cells vs. benign cells in vitro (all *p* < 0.001). *NFE2L2* silencing showed a higher expression of *ESR1* in the *NFE2L2*-downregulated cancer cell lines OVCAR3 (*p* = 0.003) and ES2 (*p* < 0.001). In the serous cancer subtype, high cytoplasmic NRF2 expression (OS, median 50.6 vs. 29.3 months; *p* = 0.04) and high ERα expression (OS, median 74.5 vs. 27.1 months; *p* = 0.002) was associated with longer overall survival as well as combined expression of both inactive cytoplasmic NRF2 and ERα in the whole cohort (median 74.5 vs. 37.7 months; *p* = 0.04). Thus, interactions of NRF2 and ERα impact survival in ovarian cancer patients and may be important factors for the response to endocrine treatment strategies.

## Figures and Tables

**Table 1:** Expression profile of NRF2 staining regarding clinical and pathological characteristics.

Parameters	*N*	Nuclear NRF2 Expression			*p*	*N*	Cytoplasmic NRF2 Expression			*p*
		Negative	Low	High			Negative	Low	High	
**Histology**										
Serous	103	0	87	16	0.02	98	0	54	44	0.001
Clear cell	11	1	7	3		11	0	11	0	
Endometrioid	20	0	18	2		19	0	12	7	
Mucinous	11	0	3	8		11	0	7	4	
**Lymph node**										
pN0/X	96	0	76	20	NS	93	0	59	34	NS
pN1	49	1	39	9		46	0	25	21	
**Distant Metastasis**										
pM0/X	141	1	112	28	NS	135	0	83	52	NS
pM1	4	0	3	1		4	0	1	3	
**Grading**										
Low	33	0	25	8	NS	33	0	16	17	0.03
High	100	1	83	16		95	0	64	31	
**FIGO**										
I/II	41	0	31	10	NS	40	0	24	16	NS
III/IV	99	0	81	18		94	0	56	38	
**Age**										
≤60 years	77	1	56	20	0.045	75	0	43	32	NS
>60 years	68	0	59	9		64	0	41	23	

**Table 2:** Correlation analysis.

*Staining*	*NRF2 Nucleus*	*NRF2 Cytoplasm*	*ERα*
**NRF2 Nucleus**			
cc	1.000	0.013	−0.019
*p*		0.88	0.82
*n*	146	138	146
**NRF2 Cytoplasm**			
cc	0.013	1.000	0.246
*p*	0.88		0.004
*n*	138	139	139
**ERα**			
cc	−0.019	0.246	1.000
*p*	0.82	0.004	
*n*	146	139	156

Immunoreactivity scores (IRS) of NRF2 and ERα staining in different compartments was correlated to each other using Spearman’s correlation analysis. cc = correlation coefficient, *p* = two-tailed significance, *n* = number of patients.

**Table 3:** Multivariate analysis.

Covariate	Coefficient (b_i_)	[HR Exp(b_i_)]	95% CI		*p*-Value
			Lower	Upper	
Histology (serous vs. other)	−0.108	0.898	0.678	1.188	0.45
Grade (low vs. high)	0.519	1.680	1.211	2.332	0.002
FIGO (I, II vs. III, IV)	0.722	2.058	1.421	2.979	0.000
Patients’ age (≤60 vs. >60 years)	0.000	1.000	1.000	1.000	0.001
NRF2 cytoplasmic/ ERα	−0.166	0.847	0.531	1.351	0.49

## Supplementary Materials

Supplementary materials can be found at http://www.mdpi.com/1422-0067/20/1/112/s1.
