# Supplementary material for: Interaction of ERα and NRF2 Impacts Survival in Ovarian Cancer Patients
Source: Int J Mol Sci. 2018 Dec 29;20(1):112. doi: 10.3390/ijms20010112 (PMC6337731; doi:10.3390/ijms20010112)
Supplement: Supplementary file 1 [file ijms-20-00112-s001.pdf]

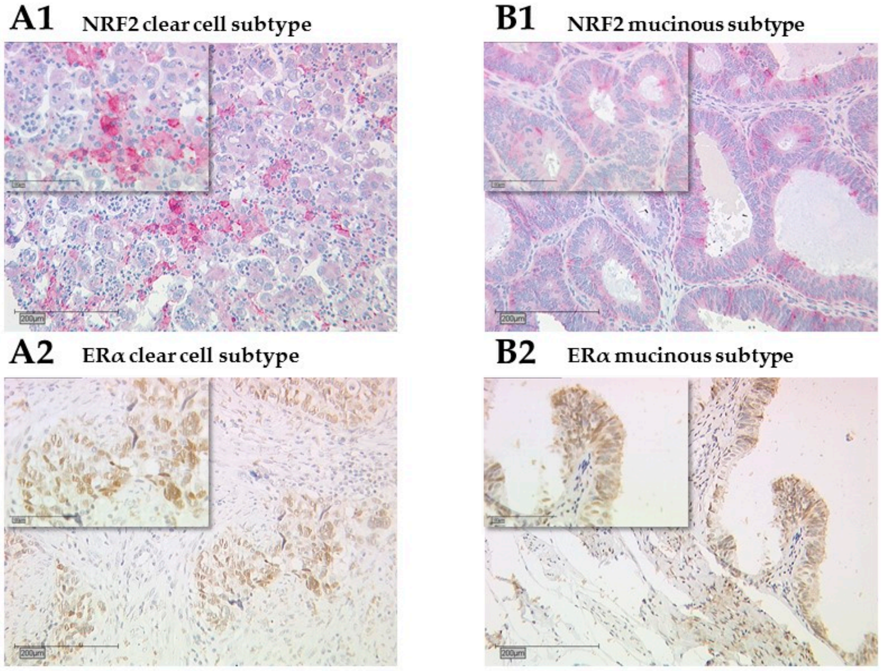

Figure S1. Detection of NRF2 (A1, B1) and ERα (A2, B2) with immunohistochemistry. High (A1) and low (B1) cytoplasmic NRF2 stains in clear cell and mucinous subtypes correspond with high (A2) and low (B2) ERα stains, respectively, in the specimens from the same individuals. NRF2 shows faint staining in the nucleus in both cases (A1, B1).

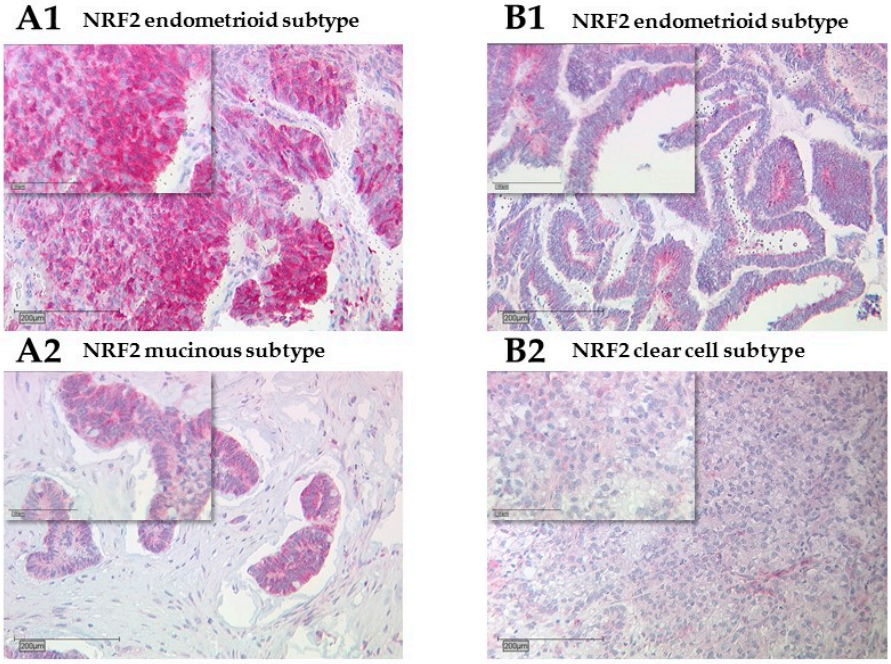

Figure S2. Detection of NRF2 with immunohistochemistry. High (A1, A2) cytoplasmic NRF2 staining in the endometrioid and mucinous EOC subtypes. Low (B1, B2) cytoplasmic NRF2 staining in the endometrioid and clear cell EOC subtypes. NRF2 shows a faint staining in the nucleus in both cases.
